# Clinical Significance of a Circulating Tumor Cell-based Classifier in Stage IB Lung Adenocarcinoma: A Multicenter, Cohort Study

**DOI:** 10.1097/SLA.0000000000004780

**Published:** 2023-01-10

**Authors:** Lijuan Ren, Xiaoming Zhong, Wei Liu, Di Xu, Yiyan Lei, Jianwen Zhou, Wenting Jiang, Qiong He, Yu Sun, Zunfu Ke

**Affiliations:** * Molecular Diagnosis and Gene Testing Center, The First Affiliated Hospital, Sun Yat-sen University, Guangzhou, P.R. China; † School of Medicine, Sun Yat-sen University, Guangzhou, P.R. China; ‡ Department of Thoracic Surgery, The Central Hospital of Wuhan, Tongji Medical College, Huazhong University of Science and Technology, Wuhan, P.R. China; § Department of Thoracic Surgery, The First Affiliated Hospital, Sun Yat-sen, University, Guangzhou, P.R. China; ∥ Institute of Precision Medicine, The First Affiliated Hospital, Sun Yat-sen University, Guangzhou, P.R. China

**Keywords:** aptamer, circulating tumor cell, prognosis, stage IB lung adenocarcinoma

## Abstract

To investigate the effectiveness of a CTC-based classifier in stratifying stage IB LUAD.

**Summary of Background Data:**

Stage IB LUADs have an approximately 70% 5-year survival rate. The clinical application of ACT is controversial due to inconsistent results in a series of trials and few useful guide biomarkers. Thus, there is a pressing need for robust biomarkers to stratify stage IB patients to define which group would most likely benefit from ACT. **Methods:** Two hundred twelve stage IB LUAD patients were enrolled and were divided into 3 independent cohorts. The aptamer-modified NanoVelcro system was used to enrich the CTCs.

**Results:**

A cutoff of <4 or >4 CTCs as the optimal prognostic threshold for stage IB LUAD was generated to stratify the patients in a 70-patient cohort into low-risk and high-risk groups. Patients with ≥ 4 CTCs in the training cohort had shorter progression-free survival (*P* < 0.0001) and overall survival (*P* < 0.0001) than patients with <4 CTCs. CTC number remained the strongest predictor of progression-free survival and overall survival even in a multivariate analysis including other clinicopathological parameters. Furthermore, a nomogram based on the CTC count was developed to predict the 3-year and 5-year survival in the training cohort and performed well in the other 2 validation cohorts (C-index: 0.862, 0.853, and 0.877).

**Conclusion:**

The presence of >4 CTCs can define a high-risk subgroup, providing a new strategy to make optimal clinical decisions for stage IB LUAD.

Lung adenocarcinoma (LUAD) is the most common histological subtype of non-small cell lung cancer (NSCLC) with a continuously high incidence.^1^ Although surgical resection is the gold standard for stage IB LUAD,^2^ 30%–40% of these patients experience relapse, which results in the failure of surgical resection; this indicates that these patients may potentially benefit from adjuvant therapy (AT) following surgery, especially adjuvant chemotherapy (ACT).^3,4^ Regrettably, numerous clinical trials have found inconsistent results in the survival benefit from AT for patients with stage IB LUAD.^5–7^ Thus, there is a pressing need for robust biomarkers to stratify the risk of stage IB patients to define which group would most likely benefit from ACT after surgery.

Currently, ACT is considered for stage IB LUAD patients with high-risk factors, including poor differentiation, wedge resection, and visceral pleura invasion, according to the National Comprehensive Cancer Network (NCCN) guidelines.^8^ However, these factors are prone to crude prognostic evaluation and unable to precisely predict cancer progression after surgery. In addition, this recommendation, belonging to category 2B, is not the first-line guideline due to a lack of high-level evidence and clinical consensus. Consequently, other classifiers or scoring systems that have not been applied clinically are being studied to predict the benefits of ACT for stage IB patients, such as the level of gene expression and the quantitative radiomic risk score.^9–11^ However, although these classifiers or scoring systems are useful to some extent, they are complex, expensive, and time-consuming. Most importantly, they can only reflect the tumor status before surgery, but cannot reveal the tumor progression after resection.

As one of the cornerstones of liquid biopsy, CTC detection has indisputable advantages of noninvasiveness, simplicity, and repeat-ability.^12^ Moreover, CTC detection is able to evaluate tumor status dynamically at any point before and after surgery.^13^ Numerous studies have revealed that the number of CTCs is an independent and effective biomarker for progression-free survival (PFS) and overall survival (OS) in patients with cancers of the lung,^14–18^ breast,^19–21^ colorectal,^22,23^ orprostate.^24–26^ Here, we explored the effectiveness of a CTC-based classifier in stratifying the risk of stage IB LUAD using an aptamer cocktail-modified NanoVelcro technology.

## Methods

### Patients

This was a prospective multicenter study conducted from October 2010 to October 2020, and CTC samples were obtained from 212 stage IB LUAD patients who underwent lobectomy or segmentectomy (Table 1). The SYSUFH cohort included 141 patients from the First Affiliated Hospital of SYSU (Guangdong, Southeast China) who were randomly divided into training (70 patients) and internal validation (71 patients) cohorts. The external validation cohort included 71 patients from the Central Hospital of Wuhan (WHCH). The 2015 tumor, node, metastasis staging system was used to classify patients with LUAD. Clinical feature data were collected through a medical record review. Patients with clinico-pathological characteristics and available follow-up information were included. The eligibility criteria included age over 20 years and histologically proven stage IB LUAD by 2 pathologists. The exclusion criteria were simultaneous pregnancy, undergoing treatment, inability to understand the study information, and loss to follow-up. Additionally, to avoid the effect of chemotherapy on the CTC count, we excluded those who had received any chemotherapy before surgery. Written informed consent was obtained from all recruited patients, and the protocols (C-084) were approved by the Medical Ethics Committee of each center.

**Table 1:** Demographics and Clinicopathological Characteristics of Training and Validation Cohorts

	Training and Validation cohorts			
Characteristic	SYSUFH Training Cohort	SYSUFH Validation Cohort	WHCH Validation Cohort	Total
No. of patients	70	71	71	212
Sex			
Male	38	34	37	109
Female	32	37	34	103
Age (yr)				
Median	59	57	60	59
Range	35–79	37–81	41–78	35–81
<60	36	39	35	110
≥60	34	32	36	102
Smoke				
Yes	13	12	15	40
No	53	58	54	165
Differentiation				
Poorly	3	4	5	12
Moderately	57	55	55	167
Well	10	12	11	33
CTC				
<4	45	39	40	124
≥4	25	32	31	88
Tumor size (cm)				
<3.5	57	63	61	181
≥3.5	13	8	10	31
Overall survival (mo)				
Median	45	58	56	52
Range	18–114	12–115	18–130	12–130
Average	51	59	61	57
Progression-free survival (mo)			
Median	36	39	39	37
Range	12–95	10–115	14–121	10–121
Average	40	43	45	43

CTC indicates circulating tumor cell; SYSUFH, The First Affiliated Hospital of Sun Yat-sen University; WHCH, The Central Hospital of Wuhan.

### CTC Enrichment and Enumeration

Peripheral blood samples (7.5 mL) were obtained from the patients within a week before surgery and preserved in ethylenedia-minetetraacetic acid-coated vacuum tubes (BD Biosciences, Franklin Lakes, NJ) for analysis. The NanoVelcro platform (Cytolumina, Los Angeles)^27^ was optimized (such as blood volume and flow rate) and used to capture the CTCs. The CTCs were captured on a NanoVelcro substrate modified by an aptamer cocktail. The captured cells were incubated with primary antibodies (CK7, Vimentin and CD45; Abcam, Cambridge, UK) at 4°C overnight. Then, secondary antibodies (Alexa Fluor 488-conjugated goat anti-mouse, CST, Danvers (Dancers, MA, USA); Alexa Fluor 555-conjugated goat anti-rabbit, CST, Danvers; and Alexa Fluor 647-conjugated goat anti-rat, Abcam) were incubated with cells for 1 hour at room temperature. Finally, 4', 6-Diamidino-2-phenylindole dihydrochloride (CST, Danvers) was added. The chips were independently analyzed by 2 experienced investigators certified in NanoVelcro technology under fluorescence microscopy at a magnification of 40 **x.** Data regarding specificity and results from healthy controls are shown in Supplementary Figure S1, http://links.lww.com/SLA/C960.

### Follow-up

According to the recommendations of the NCCN, patients were monitored every 6 months, followed by contrast-enhanced spiral computed tomography at 12 and 24 months, followed by yearly examinations, including chest computed tomography and related items.^28^

The OS and PFS were calculated by a researcher blinded to the study. OS refers to the time from random assignment to death due to any cause (or the last follow-up time for patients lost to follow-up; patients who were still alive at the end of the study were considered to be at the end of the follow-up). PFS refers to the time from randomization to the first tumor progression or death.

### Statistical Analysis

Assuming the statistical requirements for 90% power and with a 2-sided test conducted at the significance level of 0.05, at least 60 samples would need to be selected to detect a hazard ratio (HR) of at least 2 in the training cohort, corresponding to a median PFS of 48 months in the “favorable” CTC group and 24 months in the “unfavorable” group. The CTC values of 70 enrolled patients in the SYSUFH training cohort were selected to build an optimal prognostic threshold value for CTCs, which was subsequently evaluated in the internal SYSUFH validation cohort and the external WHCH validation cohort.

Statistical analysis was conducted using the “survminer,” “rms,” “survival,” “dplyr,” “foreign,” “survcomp,” “nomogram Formula,” and “pROC” packages built for R (version 4.0.2). IBM SPSS (version 25) and X-tile software (version 3.6.1) were also used to perform statistical analysis, where a 2-sided P-value < 0.05 was regarded as statistically significant. X-tile software used the Mantel-Cox test to validate the optimum cut-off value. The Chi-square test, Fisher exact test, or Spearman rank test was used to compare the correlation between CTC count and clinicopathological features. Univariate Cox and multivariable Cox proportional hazards regression were performed to analyze whether the clinical parameters were independent risk factors for PFS and OS. The log-rank test was used to compare the survival curves of the different CTC groups. A nomogram for stage IB LUAD was built to predict the 3-year and 5-year survival probabilities. The concordance index and calibration plots with bootstrap samples were used to assess the performance of the nomogram.

## Results

### Patient Demographics

From October 2010 to October 2020, 223 patients with stage IB LUAD were enrolled in the study. Eleven patients were excluded—6 as a result of poor blood quality and 5 as a result of loss to follow-up, leaving 212 evaluable patients (Supplementary Figure S2, http://links.lww.com/SLA/C960). The clinical characteristics of the 212 patients in the training and validation cohorts are listed in Table 1. For all patients, the median age was 59 years (range, 35-81 years). The number of CTCs detected in 7.5 mL of blood ranged from 0 to 13. In total, 94.3% (200/212) had at least 1 CTC, and 41.5% (88/212) had ≥4 CTCs. The median PFS time was 37 months (range, 10–121 months). The median OS time was 52 months (range, 12–130 months). SPSS was used to randomly assign a total of 141 patients from SYSUFH to the training cohort (70 patients) and the internal validation cohort, and 71 patients from WHCH were divided into the external cohort.

### Establishing the Best Prognostic Cutoff Value for CTCs

The Kaplan-Meier method and log-rank test were used to systematically analyze the survival estimation performance of a series of baseline CTC numbers in the training cohort comprising 70 patients. The thresholds were tested commencing at 1 CTC and increasing by 1 until 10 CTCs to compare the HRs and differences. Moreover, X-tile software was applied to validate the CTC threshold, and the highest *x* ^*2*^ value represented the optimal cutoff value appearing at the brightest pixel (green or red). Kaplan-Meier curves showed that there were significant differences in both the PFS and OS between the low (n = 45) and high (n = 25) subsets (HR = 5.10 and 10.18; 95% CI = 2.37–10.96 and 4.60–22.51; *P* < 0.001; Supplementary Figure S3, http://links.lww.com/SLA/C960). Consistently, the optimal cutoff value of CTCs generated from both methods was 4 CTCs per 7.5 mL, which showed the most significant difference in the PFS and OS estimation (HR = 5.1 and 10.0, 95% CI = 2.4–11.0, and 4.6–23.0; *P* < 0.001; Supplementary Tables S1 and S2, http:// links.lww.com/SLA/C960). This cutoff value was used as a classifier in the following clinical analysis.

**Figure 1:**
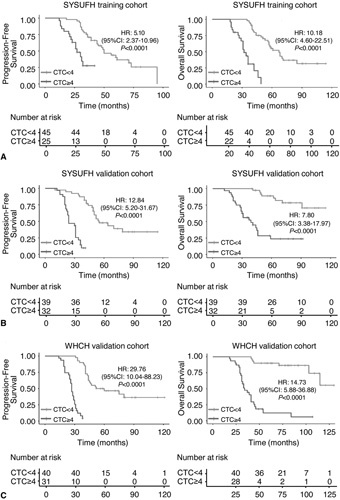
Kaplan-Meier curves for predicting progression-free survival and overall survival according to the cutoff value of 4 CTCs per 7.5 mL of blood in the 3 different cohorts. A, SYSUFH training cohort;B, internal SYSUFH validation cohort;C, external WHCH validation cohort. CI indicates confidence interval; CTC, circulating tumor cell; HR, hazard ratio; SYSUFH, First Affiliated Hospital of Sun Yat-sen University; WHCH, Wuhan Central Hospital.

To evaluate the reproducibility and validity of the CTC-based classifier, we performed internal and external validation using 2 independent data sets. The patients in these 2 cohorts were classified into low-risk and high-risk groups with the same cutoff value used in the SYSUFH training cohort (≥4 CTCs). In all 3 cohorts, patients in the high-risk group had markedly shorter PFS and OS times than those in the low-risk group (HR = 5.10 and 10.18, 12.84, and 7.80, and 29.76 and 14.73, respectively; *P* < 0.001; Fig. 1), which is consistent with the findings in the whole cohort (HR = 10.11 and 6.92; *P* < 0.001; Supplementary Figure S4, http://links.lww.com/ SLA/C960).

### Correlations Between Preoperative CTC Levels and Clinicopathological Characteristics

The correlations between CTC levels and clinical characteristics are provided in Supplementary Table S3, http://links.lww.com/ SLA/C960.. Based on the optimal prognostic CTC cutoff value (≥4 CTCs per 7.5 mL of blood), there was a significant correlation between CTC count and age (*P* < 0.001). Among 102 patients aged over 60years, 55 (53.9%) had at least 4 CTCs in 7.5 mL of blood, which is markedly higher than that (30%, 33/110) in patients aged <60 years. We did not observe any relationship between >4 CTCs and other clinical characteristics, including sex, smoking status, and tumor size. Meanwhile, no association was found between total CTC number and sex, smoking, differentiation, tumor size, and driver gene status (EGFR, K-ras mutation, and ALK rearrangement) (*P* > 0.05, Supplementary Figure S5, http://links.lww.com/SLA/C960). Among patients with stage IB LUAD, the micropapillary predominant subtype showed significantly more CTCs than the solid-predominant subtype (P = 0.007, Supplementary Figure S6, http://links.lww.com/ SLA/C960).

### Univariate and Multivariate Analyses of Clinical Characteristics

Univariate and multivariate Cox proportional hazards regression analyses were used to identify potential independent prognostic factors (Tables 2 and 3). In the univariate analysis, the clinical factors significantly associated with survival were CTC level and age. The highest HRs for both PFS and OS were defined by the CTC count (≥4). In the multivariate analysis, CTC count (≥4) remained an independent prognostic factor for the PFS (P < 0.001) and OS (P < 0.001) (Table 3), which is in line with the results of the univariate analysis.

**Table 2:** Univariate Analysis for Clinicopathological Characteristics in Stage IB LUAD Patients

	PFS			OS		
Risk Factor	HR	95% CI	*P*	HR	95% CI	*P*
SYSUFH Training Cohort (n = 70)						
Sex (male vs female)	1.2	0. 6–2.2	0.61	1.2	0.7–2.3	0.49
Age (<60 vs ≥60)	3.1	1.6–5.8	<0.001	2.9	1.6–5.4	<0.001
Smoke (yes vs no)	0.8	0.4–1.8	0.61	0.7	0.3–1.6	0.39
Differentiation	1.1	0.4–2.5	0.91	1.0	0.4–2.2	0.90
CTC (<4 vs ≥4)	5.1	2.4 to 11.0	<0.001	10.0	4.6-23.0	<0.001
SYSUFH Validation Cohort (n = 71)						
Sex (male vs female)	0.9	0.5-1.6	0.68	0.8	0.4-1.7	0.57
Age (<60 vs ≥60)	1.3	0.8–2.4	0.31	4.4	2.0–9.6	<0.001
Smoke (yes vs no)	1.4	0.7–2.9	0.36	1.5	0.6–3.7	0.36
Differentiation	0.7	0.4–1.1	0.10	0.8	0.4–1.6	0.46
CTC (<4 vs ≥4)	13.0	5.2–32.0	<0.001	7.8	3.4–18.0	<0.001
WHCH Validation Cohort (n = 71)						
Sex (male vs female)	1.6	0.9–2.7	0.10	1.6	0.8–3.4	0.18
Age (<60 vs ≥60)	2.0	1.1–3.5	0.016	3.9	1.7–8.8	0.001
Smoke (yes vs no)	1.8	1.0–3.4	0.063	1.4	0.6–3.0	0.40
Differentiation	0.8	0.4–1.5	0.51	0.7	0.3–1.5	0.38
CTC (<4 vs ≥4)	30.0	10.0–88.0	<0.001	15.0	5.9–37.0	<0.001
All (n = 212)						
Sex (male vs female)	1.2	0.8–1.6	0.37	1.3	0.9–1.9	0.24
Age (<60 vs ≥60)	2.0	1.4–2.7	<0.001	3.4	2.3–5.1	<0.001
Smoke (yes vs no)	1.3	0.9–2.0	0.19	1.2	0.7–1.9	0.52
Differentiation	0.7	0.5–1.1	0.12	0.8	0.5–1.2	0.26
CTC (<4 vs ≥4)	10.0	6.4–16.0	<0.001	6.9	4.6–10.0	<0.001

CTC indicates circulating tumor cell; HR, hazard ratio; LUAD, lung adenocarcinoma; OS, overall survival; PFS, progression-free survival; SYSUFH, The First Affiliated Hospital of Sun Yat-sen University; WHCH, The Central Hospital of Wuhan.

**Table 3:** Multivariate Cox Regression Analysis for Clinicopathological Characteristics in Stage IB LUAD Patients

	PFS		OS	
	HR (95% CI)	P	HR (95% CI)	P
SYSUFH Training Cohort (n = 70)				
Sex (male vs female)	1.02 (0.47–2.23)	0.965	0.71 (0.32–1.60)	0.392
Age (<60 vs ≥60)	2.93 (1.32–6.50)	0.008	3.17 (1.45–6.90)	0.004
Smoke (yes vs no)	1.32 (0.50–3.52)	0.579	1.14 (0.43–3.00)	0.799
Differentiation				
Poorly	Ref		Ref	
Moderately	0.24 (0.07–0.88)	0.031	0.52 (0.12–2.30)	0.386
Well	0.67 (0.15–3.07)	0.604	0.95 (0.16–5.60)	0.955
CTC (<4 vs ≥4)	4.63 (2.04–10.47)	<0.001	10.71 (4.32–26.60)	<0.001
SYSUFH Validation Cohort (n = 71)				
Sex (male vs female)	1.02 (0.50–2.10)	0.962	0.71 (0.27–1.80)	0.484
Age (<60 vs ≥60)	1.30 (0.70–2.40)	0.407	3.62 (1.57–8.30)	0.003
Smoke (yes vs no)	0.82 (0.33–2.00)	0.673	1.09 (0.35–3.30)	0.886
Differentiation				
Poorly	Ref		Ref	
Moderately	0.76 (0.21–2.80)	0.685	0.64 (0.07–5.50)	0.685
Well	0.22 (0.05–1.00)	0.057	0.28 (0.03–3.20)	0.311
CTC (<4 vs ≥4)	18.10 (6.59–49.70)	<0.001	6.58 (2.68–16.20)	<0.001
WHCH Validation Cohort (n = 71)				
Sex (male vs female)	1.30 (0.64–2.50)	0.496	1.36 (0.56–3.30)	0.495
Age (<60 vs ≥60)	2.90 (1.51–5.50)	0.001	3.41 (1.45–8.00)	0.005
Smoke (yes vs no)	2.60 (1.18–5.90)	0.019	0.90 (0.35–2.30)	0.828
Differentiation				
Poorly	Ref		Ref	
Moderately	2.00 (0.59–6.70)	0.266	1.56 (0.44–5.60)	0.495
Well	1.90 (0.48–7.50)	0.357	0.74 (0.15–3.70)	0.714
CTC (<4 vs ≥4)	49.20 (14.78–63.50)	<0.001	14.81 (5.77–38.00)	<0.001
All (n = 212)				
Sex (male vs female)	1.02 (0.69–1.50)	0.913	1.06 (0.67–1.70)	0.813
Age (<60 vs ≥60)	1.77 (1.24–2.50)	0.002	3.00 (1.94–4.60)	<0.001
Smoke (yes vs no)	1.39 (0.87–2.20)	0.172	0.99 (0.58–1.70)	0.975
Differentiation				
Poorly	Ref		Ref	
Moderately	0.72 (0.37–1.40)	0.341	1.00 (0.43–2.30)	0.998
Well	0.56 (0.25–1.20)	0.156	0.64 (0.23–1.80)	0.382
CTC (<4 vs ≥4)	10.61 (6.60–17.10)	<0.001	6.70 (4.32–10.40)	<0.001

CTC indicates circulating tumor cell; HR, hazard ratio; LUAD, lung adenocarcinoma; OS, overall survival; PFS, progression-free survival; SYSUFH, The First Affiliated Hospital of Sun Yat-sen University; WHCH, The Central Hospital of Wuhan.

### Subsequent Intense Therapy Based on ≥4 CTCs per 7.5 mL of Blood Leads to a Better Clinical Outcome

**Figure 2:**
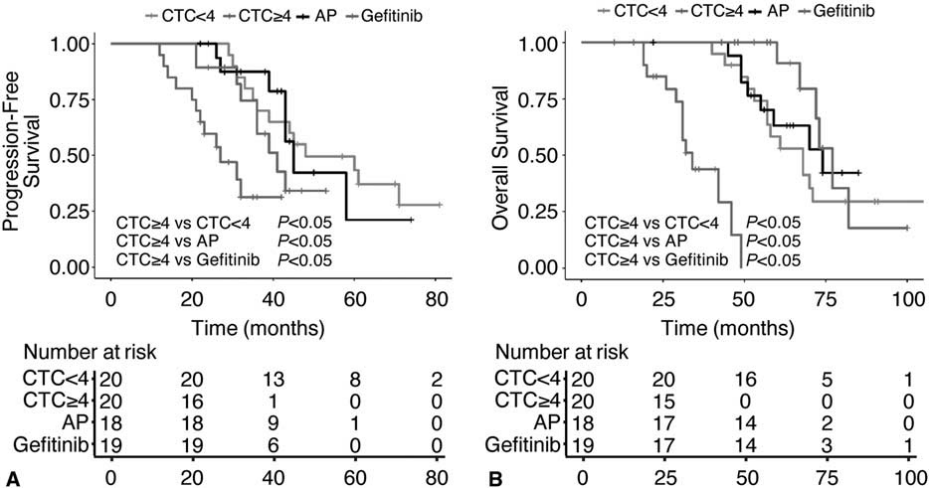
Kaplan-Meier curves for predicting progression-free survival and overall survival in patients with and without adjuvant therapy. A, Progression-free survival;B, overall survival. For blue line, patients were detected with CTC <4 and received surgery only. For purple line, patients were detected with CTC ≥4 and received surgery only. For black line, patients were detected with CTC ≥4 and then received AP-chemotherapy scheme (pemetrexed, disodium, and cisplatin) following surgery. For red line, patients were detected with CTC ≥4 and then received Gefitinib-targeted therapy following surgery. CTC indicates circulating tumor cell.

To validate the value of the CTC-based classifier in clinical practice, data from another 37 stage IB LUAD patients, of whom 18 received AP-chemotherapy regimens (pemetrexed disodium and cisplatin) and 19 received gefitinib (patients with EGFR sensitive mutations) after radical surgery, were collected using the same inclusion criteria. As shown in Figure 2, both PFS and OS were poor in patients with ≥4 CTCs and no AT than those in patients with <4 CTCs. However, in patients with ≥4 CTCs and AT, both the PFS and OS were better than those in patients with ≥4 CTCs and no AT (P < 0.05), and similar to those in patients with <4 CTCs, indicating the potential benefit of further AT after surgery.

### Analysis of Stratification by the CTC-based Prognostic Classifier

**Figure 3:**
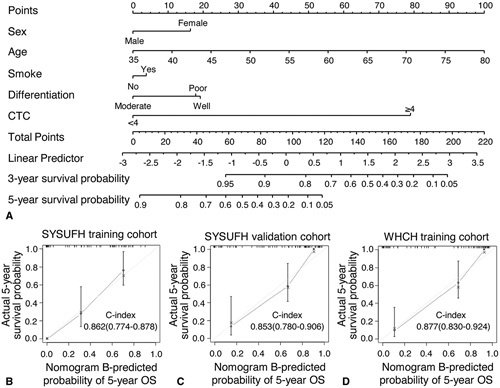
Nomogram B (including CTC level) for predicting the 3- and 5-yr survival probabilities in patients with stage IB LUAD and calibration curves for testing the stability of nomogram. A, Nomogram B was based on the multivariate analysis results of the SYSUFH training cohort. B, Calibration curves of SYSUFH training cohort. C, Calibration curves of internal SYSUFH validation cohort. D, Calibration curves of external WHCH validation cohort. C-index indicates concordance index;CTC, circulating tumor cell;LUAD, lung adenocarcinoma; SYSUFH, First Affiliated Hospital of Sun Yat-sen University; WHCH, Wuhan Central Hospital.

**Figure 4:**
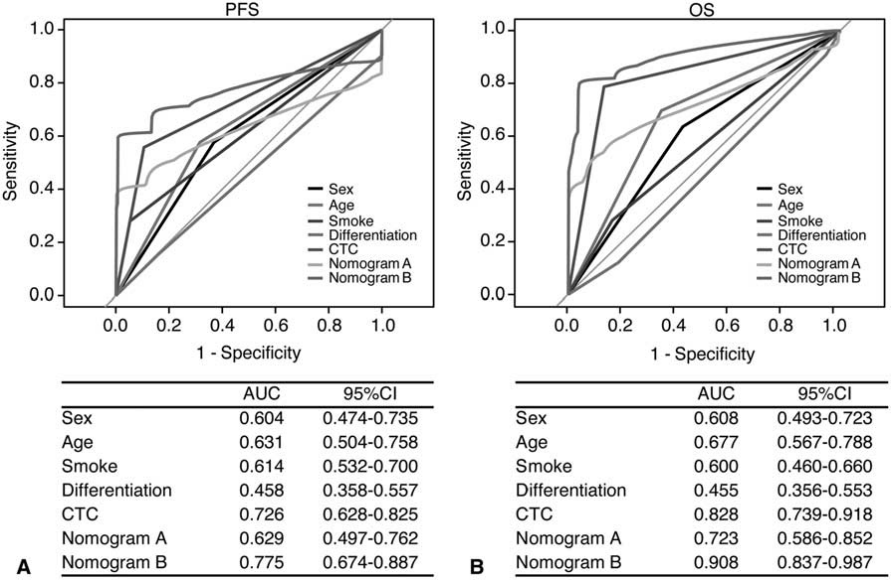
Time-dependent ROC curves for assessing the sensitivity and specificity of nomogram A, nomogram B, CTC and other factors in predicting PFS and OS in all 212 patients with stage IB LUAD. A, ROC curves predicting PFS. B, ROC curves predicting OS. Nomogram A consists of sex, age, smoking, and differentiation. Nomogram B consists of sex, age, smoking, differentiation and CTC. AUC indicates area under the curve; CI, confidence interval; CTC, circulating tumor cell; LUAD, lung adenocarcinoma; OS, overall survival; PFS, progression-free survival; ROC indicates, receiver operator characteristic.

When further survival analysis was carried out for subsets of all patients with different clinical variables based on the CTC classifier, the CTC count was still a statistically and clinically significant prognostic signature. As shown in Supplementary Figure S7 and S8, http://links.lww.com/SLA/C960, in different subsets, the cutoff value of 4 CTCs per 7.5 mL of blood can well classify them into high-risk and low-risk groups in both PFS and OS (males, *P* < 0.001; females, *P* < 0.001; age < 60 years, *P* < 0.001; age ≥ 60 years, *P* < 0.001; no smoking, *P* < 0.001; smoking, *P* < 0.001; poorly differentiated, *P* < 0.05; moderately differentiated, *P* < 0.001; well differentiated, *P* < 0.01, respectively). Based on clinicopathological covariates, such as sex, age, smoking, and differentiation, we constructed a clinically applicable nomogram, nomogram A, which was used to evaluate 3-year or 5-year survival probability in the 3 cohorts (Supplementary Figure S9, http://links. lww.com/SLA/C960). Because the CTC count exhibited the highest C-index among the covariates (C-index 0.708, 95% CI 0.685-0.785; *P* < 0.001; Supplementary Table S4, http://links.lww.com/SLA/ C960), we integrated the same into nomogram A and then built a final nomogram, named nomogram B. Calibration plots demonstrated that nomogram B performed better than nomogram A in the 3 cohorts (C-index 0.862, 95% CI 0.774–0.878 for the SYSUFH training cohort; C-index 0.853, 95% CI 0.780-0.906 for the SYSUFH validation cohort; and C-index 0.877, 95% CI 0.830– 0.924 for the WHCH validation cohort; Fig. 3), which was further confirmed by ROC analysis. As shown in Figure 4, nomogram B as a predictor exhibited higher sensitivity and specificity than other factors in both the PFS and OS (AUC = 0.775 and 0.908, 95% CI = 0.674-0.887 and 0.837–0.987, respectively).

## Discussion

Current NCCN guidelines^8^ indicate a controversy over the application of ACT in patients with stage IB LUAD. In this study, we demonstrated for the first time the effect of CTC count on the prognosis of stage IB LUAD; and the CTC-based prognostic classifier may provide a new strategy for making optimal clinical decisions for stage IB patients. Patients at a high risk of recurrence, even those with tumors clinicopathologically defined as well-differentiated cancers, need to be screened for ACT to reduce recurrence and prolong survival. Patients at a low risk of recurrence, even those with tumors clinicopathologically defined as poorly differentiated cancers, need to be screened to protect them from the toxicity of ACT.

As the cornerstone of liquid biopsy, ctDNA is a potential biomarker for real-time monitoring of early lung cancer.^29^ However, the majority of the biological mutations detected probably came from cell-free DNA released from hematopoietic lineage cells.^30^ Even if it has been recently addressed, follow-up tests are needed to verify whether the mutations detected are specific for a cancer cell population.^31^ In contrast, it is undoubtedly more ideal for isolating CTCs and analyzing their role in predicting the prognosis of tumors. CTCs are the seeds of distant metastasis of cancer,^32^ and have been confirmed in many studies to be related to the poor prognosis of various cancers, including lung,^16–18^ breast,^19,33^ colorectal^34^ and prostate cancer.^35^ In our study, 94.3% of patients with stage IB LUAD contained ≥1 CTC in 7.5 mL of blood, which was significantly higher than the positive rates reported by Hofman^36^ and Blackhall^16^ using traditional EpCAM-mediated CTC capture methods. This further demonstrated that EpCAM-dependent CTC enrichment is insufficient for the comprehensive characterization of CTC heterogeneity because tumor heterogeneity leads to the proliferation of polyclonal cancer cells with distinct phenotypes.^37^ In contrast, our cell-specific aptamers are ideal CTC agents, especially for cancer cells that lack available antibodies.^38^ Some studies previously reported the optimal cutoff of CTCs in colon cancer (>3 CTCs/ 7.5 mL),^39^ gestational choriocarcinoma (≥6CTCs/7.5mL),^40^ metastatic breast cancer, and prostate cancer (≥5CTCs/7.5mL).^35,41^ In our training cohort, the survival difference in the 36-month PFS and OS reached a maximum for a cutoff of ≥4 CTCs, which could distinguish between patients with unfavorable and favorable prognoses. When dichotomizing patient groups for prognosis, clinicians should be careful about the application of low threshold values because it could lead to a risk of stratifying patients into the wrong prognostic subgroup.^42,43^ Therefore, a high threshold of 4 CTCs determined in our study would reduce the risk of incorrectly assigning patients to the wrong risk groups.

Naoki et al reported that the micropapillary pattern was a significant predictor of recurrence in resected stage I LUAD.^44^ We also noted that all of 4 patients with micropapillary predominant subtype showed ≥4 CTCs/7.5 mL, which was significantly higher than that in the solid predominant subtype, further demonstrating that patients with predominant micropapillary should receive ACT after surgery.^45^ Multivariate Cox regression analysis showed that CTC count was an independent risk factor for PFS and OS in stage IB LUAD, but did not indicate that pathological differentiation was an independent risk factor, which showed that the accuracy of CTC count in survival prediction was higher than that of pathological differentiation. Thus, it is more beneficial to use CTC count to guide postoperative individual ACT, which can reduce the economic burden of patients with poorly differentiated tumors, but with good prognosis and improve their quality of life. This finding also suggests that patients with high CTC levels who have well-differentiated tumors should undergo postoperative AT to reduce the possibility of recurrence and prolong survival.

Before this study, several models integrated multiple biomark-ers into a signature to substantially improve the clinical prognostic predictive value in NSCLC.^46–48^ Owing to the complexity of genome-wide technologies, unintegrated high-throughput biomarkers, and inappropriate statistical methods, their clinical application has been deterred by great limitations. Recently, a retrospective study demonstrated that the quantitative radiomic risk score can predict the added benefit of ACT following surgery for patients with stage IB LUAD.^49^ However, the accuracy of the score depends on the operator's experience and subjective judgment to some extent. As a single marker with the characteristics of noninvasiveness and repeatability, our CTC-based classifier is both more feasible and inexpensive than the prognostic signatures from previous studies.

The CTC-based classifier can accurately stratify patients with stage IB LUAD. The prognostic accuracy of the CTC-based classifier was similar among the 3 cohorts, indicating its reproducibility regardless of the clinical center.

In addition, we built a nomogram to predict individual patients' recurrence risk, and its performance was well verified in all validation cohorts. Traditional nomograms use clinical prognostic factors, such as sex, age, smoking, and differentiation, whereas CTC levels can reflect the biological status of primary tumors,^13^ and elevated CTC counts indicate poor survival in LUAD. Moreover, integrating CTC counts into this nomogram increased its predictive accuracy. Thus, our nomogram will pave the way for developing a simple and accurate method for prognostic prediction in stage IB LUAD.

There are several limitations to the present study. First, the classifier was developed based on an analysis of Chinese patients, limiting its immediate clinical application worldwide. Second, although the optimum CTC threshold can stratify patients into high-risk and low-risk subgroups, whether the former benefit from AT needs to be confirmed in a further clinical trial. Third, liquid biopsy is in a period of vigorous development,^50^ and currently, there is still no perfect method for detecting CTCs in lung cancer.

In conclusion, the newly developed CTC-based classifier can categorize stage IB LUAD patients into low-risk and high-risk groups, the latter of which may benefit from other treatments, such as ACT. CTC detection will facilitate patient consultation, adjustment of follow-up protocols, and patient selection for optimum adjuvant trial designs.

## Supplementary Material
