# Supplementary material for: Clinical Significance of a Circulating Tumor Cell-based Classifier in Stage IB Lung Adenocarcinoma: A Multicenter, Cohort Study
Source: Ann Surg. 2023 Jan 10;277(2):e439–48. doi: 10.1097/SLA.0000000000004780 (PMC9831033; doi:10.1097/SLA.0000000000004780)
Supplement: Supplementary file 1 [file sla-277-e439-s001.pdf]

Supplementary data:
Supplementary Tables: 4
Supplementary Figures: 9

Supplementary Table S1. Establishment of CTC Cutoff Value in Training Cohort

CTC cutoff	Group*	No. in each group	36-Month PFS (%)	Survival difference (%)	HR	95%CI	*P*
1	0	3	100.0	52.2	1.7	0.4-7.1	0.4700
	≥ 1	67	47.8				
2	0-1	21	85.7	51.0	2.2	1.1-4.4	0.0210
	≥ 2	49	34.7				
3	0-2	30	80.0	52.5	4.7	2.1-11.0	0.0002
	≥ 3	40	27.5				
4	0-3	45	73.3	65.3	5.1	2.4-11.0	.0000
	≥ 4	25	8.0				
5	0-4	56	60.7	53.6	5.1	2.1-12.0	0.0003
	≥ 5	14	7.1				
6	0-5	62	56.5	-	6.1	1.8-20.0	0.0032
	≥ 6	8	-				
7	0-6	64	54.7	-	11.0	3.2-39.0	0.0002
	≥ 7	6	-				
8	0-7	67	52.2	-	13.0	2.5-65.0	0.0023
	≥ 8	3	-				
9	0-8	67	52.2	-	13.0	2.5-65.0	0.0023
	≥ 9	3	-				
10	0-9	69	50.7	-	42.0	3.7-480.0	0.0025
	≥ 10	1	-				

*Split into below and above different CTC cutoffs in rows.
P indicates significance according to Cox proportional hazards regression.
CTC indicates circulating tumor cell; HR, hazard ratio; PFS, progression-free survival; CI, confidence interval.

Supplementary Table S2. Establishment of CTC Cutoff Value in Training Cohort

CTC cutoff	Group	No. in each group	36-Month OS (%)	Survival difference (%)	HR	95%CI	*P*
1	0	3	100.0	28.4	4.9	0.7-36.0	0.1200
	≥ 1	67	71.6				
2	0-1	21	100	38.8	5.0	2.3-11.0	.0000
	≥ 2	49	61.2				
3	0-2	30	96.7	41.7	8.7	4.0-19.0	.0000
	≥ 3	40	55.0				
4	0-3	45	97.8	69.8	10.0	4.6-23.0	.0000
	≥ 4	25	28.0				
5	0-4	56	83.9	60.7	8.2	3.4-20.0	.0000
	≥ 5	14	28.6				
6	0-5	62	80.6	68.1	10.0	3.9-28.0	.0000
	≥ 6	8	12.5				
7	0-6	64	78.1	61.4	9.6	3.5-27.0	.0000
	≥ 7	6	16.7				
8	0-7	67	76.1	-	47.0	7.5-290.0	.0000
	≥ 8	3	-				
9	0-8	67	76.1	-	47.0	7.5-290.0	.0000
	≥ 9	3	-				
10	0-9	69	73.9	-	22.0	2.3-220.0	0.0070
	≥ 10	1	-				

*Split into below and above different CTC cutoffs in rows.

P indicates significance according to Cox proportional hazards regression.

CTC indicates circulating tumor cell; HR, hazard ratio; OS, overall survival; CI, confidence interval.

Supplementary Table S3. The Associations Between Different CTC Levels and Clinicopathological Characteristics in 212 Stage IB LUAD Patients

	**Patients with CTCs (%)**							
Characteristic	≥1	≥2	≥3	≥4	≥5	≥6	≥7	≥10
All (n=212)	94.3	89.6	56.6	41.5	20.8	10.4	7.5	1.4
**Sex**								
Male	94.5	76.1	56.0	39.4	22.9	14.7	11.0	1.8
Female	94.2	74.8	57.3	41.7	18.4	5.8	3.9	1.0
P	1	0.874	0.890	1	0.499	0.043	0.068	1
**Age** (years)								
<60	90.9	63.6	42.7	30.0	10.9	3.6	2.7	0.0
≥60	98.0	88.2	71.6	53.9	31.4	17.6	12.7	2.9
P	0.035	<0.001	<0.001	<0.001	<0.001	0.001	0.008	0.110
**Smoke**								
Yes	95.0	82.5	55.0	42.5	27.5	12.5	10.0	5.0
No	93.9	72.7	56.4	41.8	18.8	9.7	6.7	0.6
P	1	0.230	1	1	0.274	0.569	0.499	0.098
**Differentiation**								
Poorly	100.0	83.3	50.0	33.3	8.3	0.0	0.0	0.0
Moderately	94.0	75.4	56.9	41.3	22.2	12.0	8.4	1.8
Well	93.9	72.7	57.6	45.5	18.2	6.1	6.1	0.0
P	<0.01	<0.01	<0.01	<0.01	<0.01	<0.01	<0.01	0.333
**Tumor size (cm)**								
<3.5	93.9	73.5	56.4	40.9	21.0	11.0	7.7	1.7
≥3.5	96.8	87.1	58.1	45.2	19.4	6.5	6.5	0.0
P	1	0.539	0.859	0.655	0.835	0.749	1	0.097

P indicates significance according to Chi-square test or Fisher's exact test.
CTC indicates circulating tumor cell; LUAD, lung adenocarcinoma.

Supplementary Table S4: The C-Index of the Five Enrolled Factors in the Nomogram in the SYSUFH Training Cohort

Factors	C-index	95% CI	*P*
Sex	0.507	0.423-0.590	0.883
Age	0.682	0.606-0.759	< .001
Smoke	0.536	0.472-0.600	0.274
Differentiation	0.530	0.464-0.560	0.374
CTC	0.708	0.685-0.785	< .001

P indicates significance according to Cox proportional hazards regression
C-index indicates concordance index; CI, confidence interval; CTC, circulating tumor cell; SYSUFH, The First Affiliated Hospital of Sun Yat-sen University.

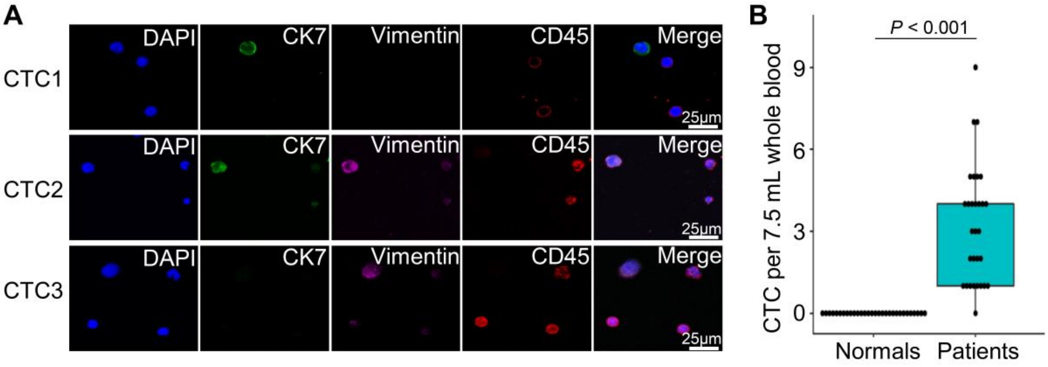

Supplementary Figure S1: Gallery of CTC images from the aptamer-modified NanoVelcro system obtained from 7.5 mL of blood from 30 healthy donors and 30 stage IB LUAD patients. A, CTC1 shows that epithelial CTCs express CK7. CTC2 shows that hybrid CTCs express CK7 and Vimentin. CTC3 shows that mesenchymal CTCs express Vimentin. B, CTC number in 7.5 mL of peripheral blood from stage IB LUAD patients was significantly higher than that in heathy controls (P<0.001). CTC indicates circulating tumor cell; LUAD, lung adenocarcinoma.

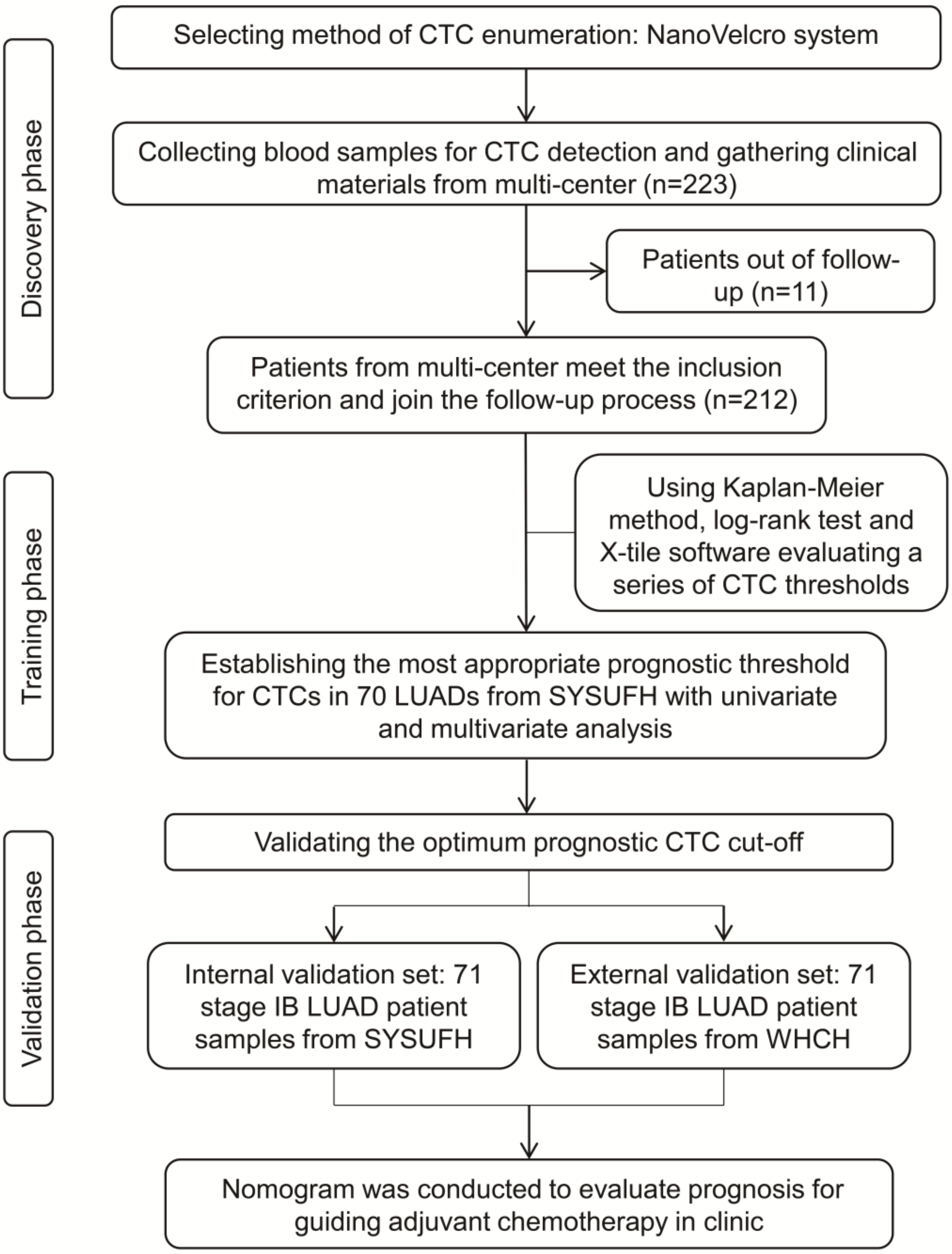

Supplementary Figure S2: Study flowchart of the development and evaluation of the CTC-based classifier in stratifying the risk of stage IB LUAD. CTC indicates circulating tumor cell; LUAD, lung adenocarcinoma; SYSUFH, First Affiliated Hospital of Sun Yat-sen University; WHCH, Wuhan Central Hospital.

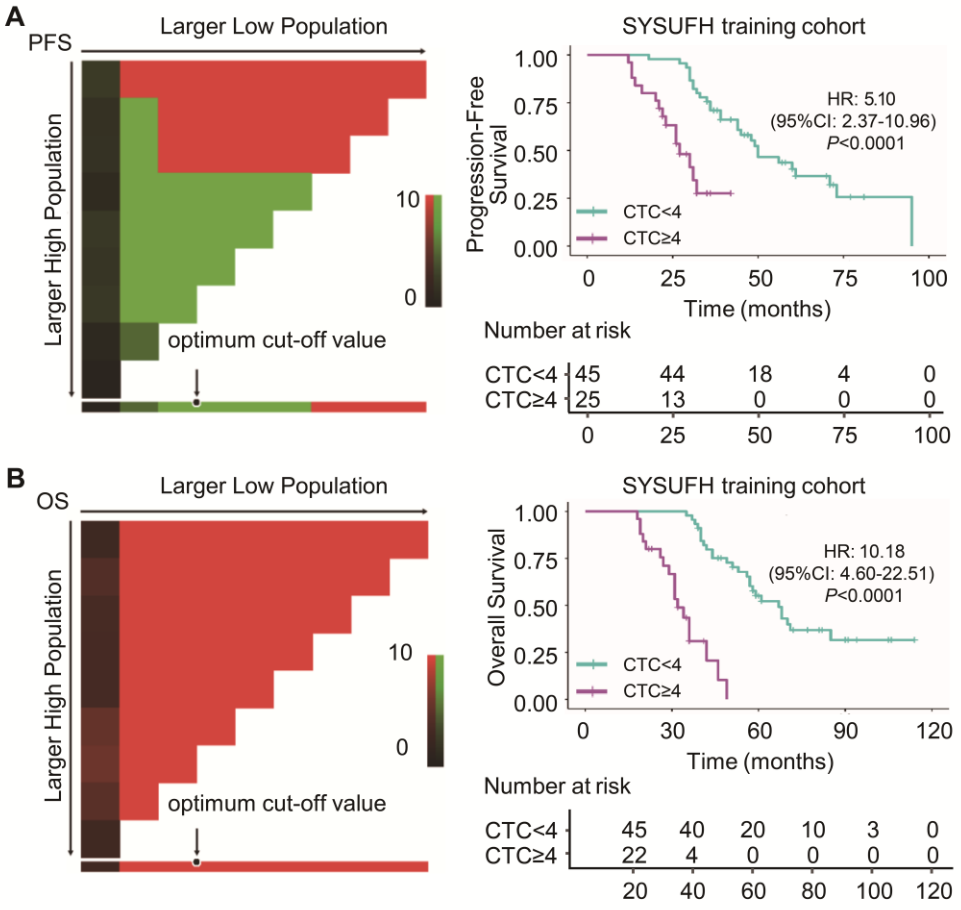

Supplementary Figure S3: The optimum cutoff value of CTC count and Kaplan-Meier curves for predicting PFS and OS in the training cohort with 70 stage IB LUAD patients. A, Progression-free survival; B, Overall survival. The optimum cutoff value was defined using the X-tile program**,** and the coloration of the plot represents the strength of the association at each division. Red represents a positive association between the risk score and PFS or OS, whereas green represents an inverse association. CTC indicates circulating tumor cell; PFS, progression-free survival; OS, overall survival; HR, hazard ratio.

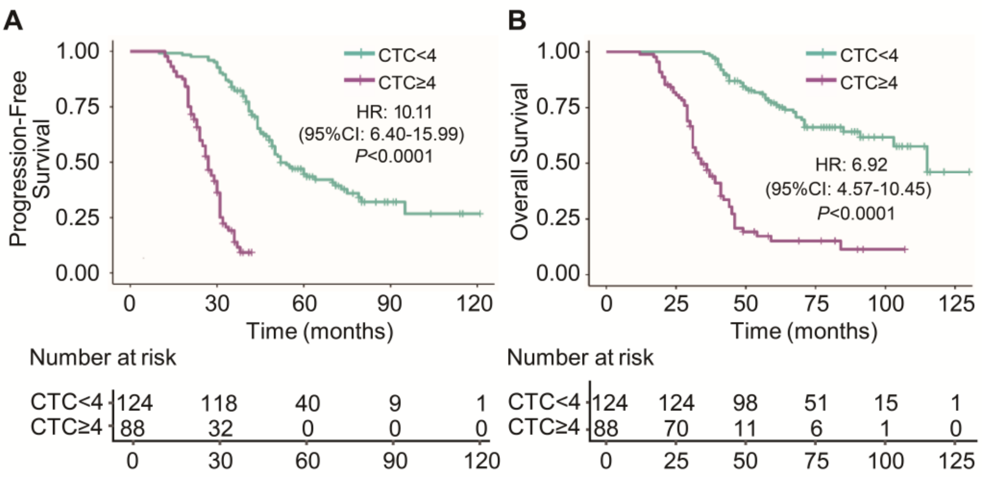

Supplementary Figure S4. Kaplan-Meier curves for predicting PFS and OS according to the cutoff value of 4 CTCs per 7.5 mL of blood in all 212 patients with stage IB LUAD. A, Progression-free survival according to the number of CTCs detected in the total patient cohort; B, Overall survival according to the number of CTCs detected in the total patient cohort. *P* indicates significance according to log Rank test. CTC indicates circulating tumor cell; PFS, progression-free survival; OS, overall survival; HR, hazard ratio.

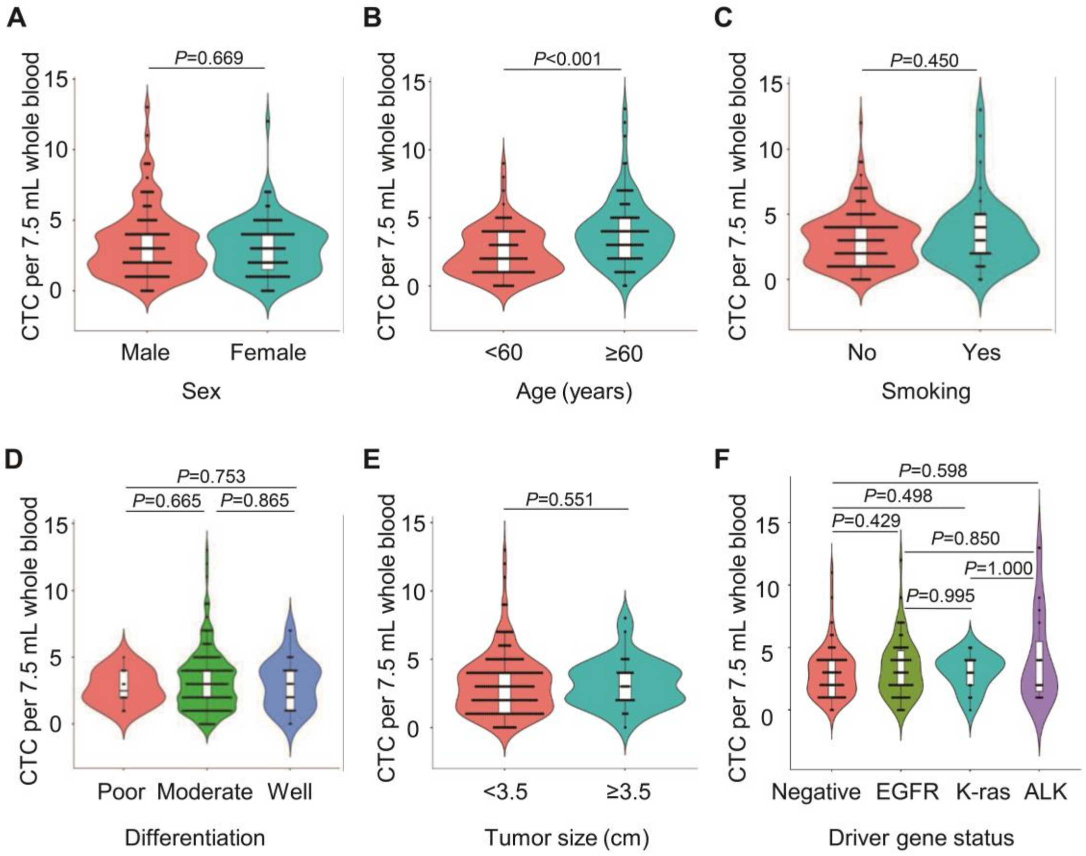

Supplementary Figure S5. Relationship between total CTC number and different clinicopathological characteristics. A, Prevalence of CTCs in 7.5 mL of blood in sex: male (n = 109) and female (n = 103). B, Prevalence of CTCs in 7.5 mL of blood in age (years): <60 (n = 110) and ≥ 60 (n= 102). C, Prevalence of CTCs in 7.5 mL of blood in status of smoking: no (n=165) and yes (n = 40). D, Prevalence of CTCs in 7.5 mL of blood in different differentiation: poor (n = 12), moderate (n = 167) and well (n = 33). E, Prevalence of CTCs in 7.5 mL of blood in different tumor size: < 3.5 cm (n = 181) and ≥ 3.5 cm (n = 31). F, Prevalence of CTCs in 7.5 mL of blood from different driver gene groups: negative (n= 65), EGRF mutation (n = 62), K-ras mutation (n = 17), ALK rearrangement (n = 15). Other 53 patients have no gene testing reports. CTC indicates circulating tumor cell.

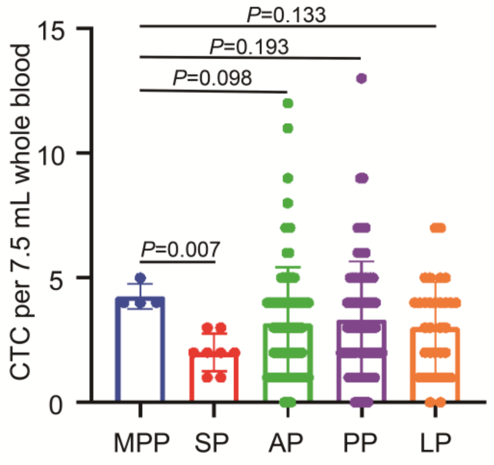

Supplementary Figure S6. Prevalence of CTCs in 7.5 mL of blood from different histologic subtypes of LUAD. MPP (n = 4), SP (n = 8), AP (n = 88), PP (n =79), LP (n = 33). n indicates the number of patients in each group. CTC indicates circulating tumor cell; MPP, Micropapillary predominant; SP, solid predominant; AP, acinar predominant; PP, papillary predominant; LP, Lepidic predominant.

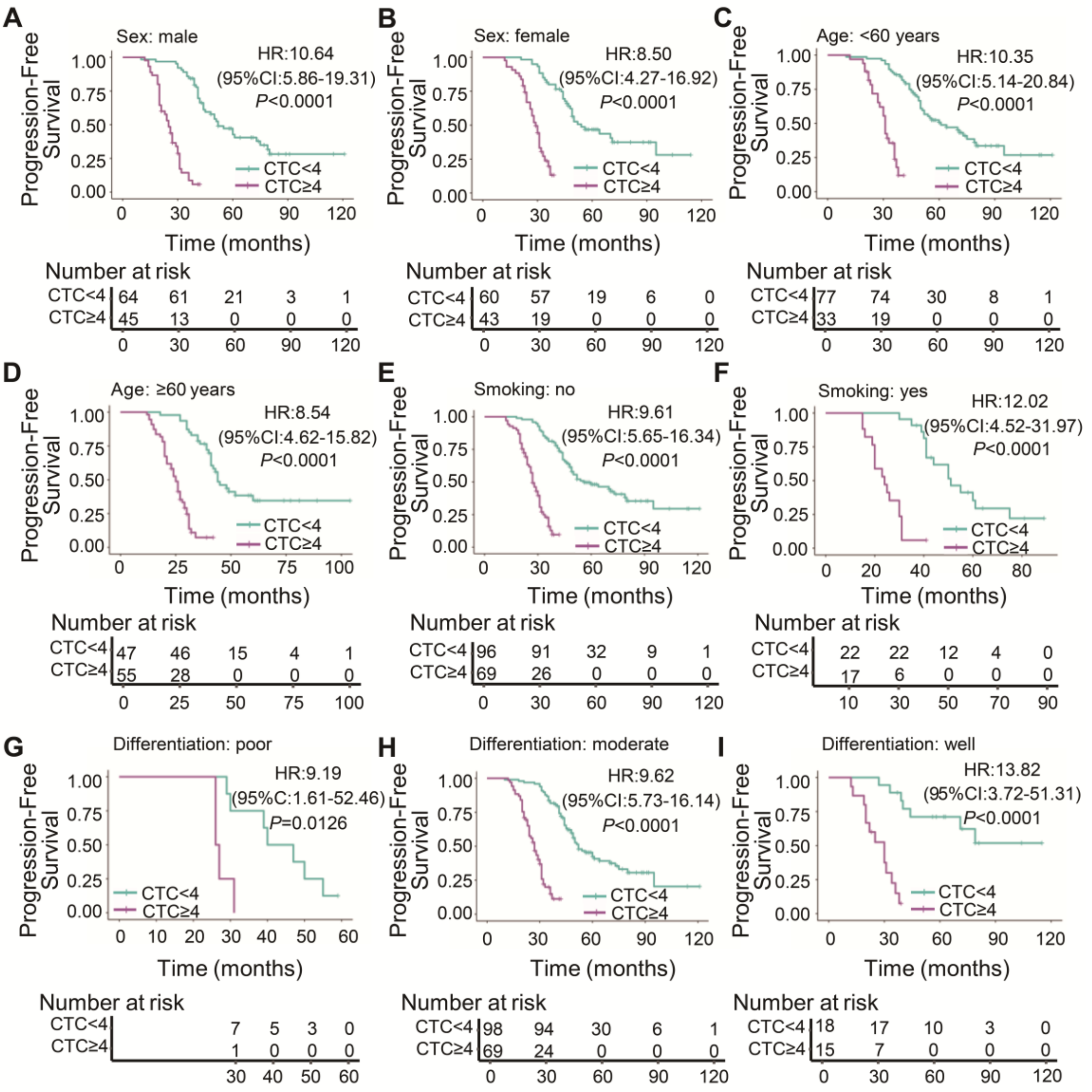

Supplementary Figure S7. Kaplan-Meier curves for predicting PFS with different clinicopathological characteristics according to the cutoff value of 4 CTCs per 7.5 mL of blood in all 212 patients. A, male with CTC<4 or CTC≥4 groups; B, female with CTC<4 or CTC≥4 groups; C, age<60 with CTC<4 or CTC≥4 groups; D, age≥60 with CTC<4 or CTC≥4 groups; E, smoking with CTC<4 or CTC≥4 groups; F, no smoking with CTC<4 or CTC≥4 groups; G, poor differentiation with CTC<4 or CTC≥4 groups; H, moderate differentiation with CTC<4 or CTC≥4 groups; I, well differentiation with CTC<4 or CTC≥4 groups. *P* indicates

significance according to log Rank test. CTC indicates circulating tumor cell; PFS, progression-free survival; HR, hazard ratio.

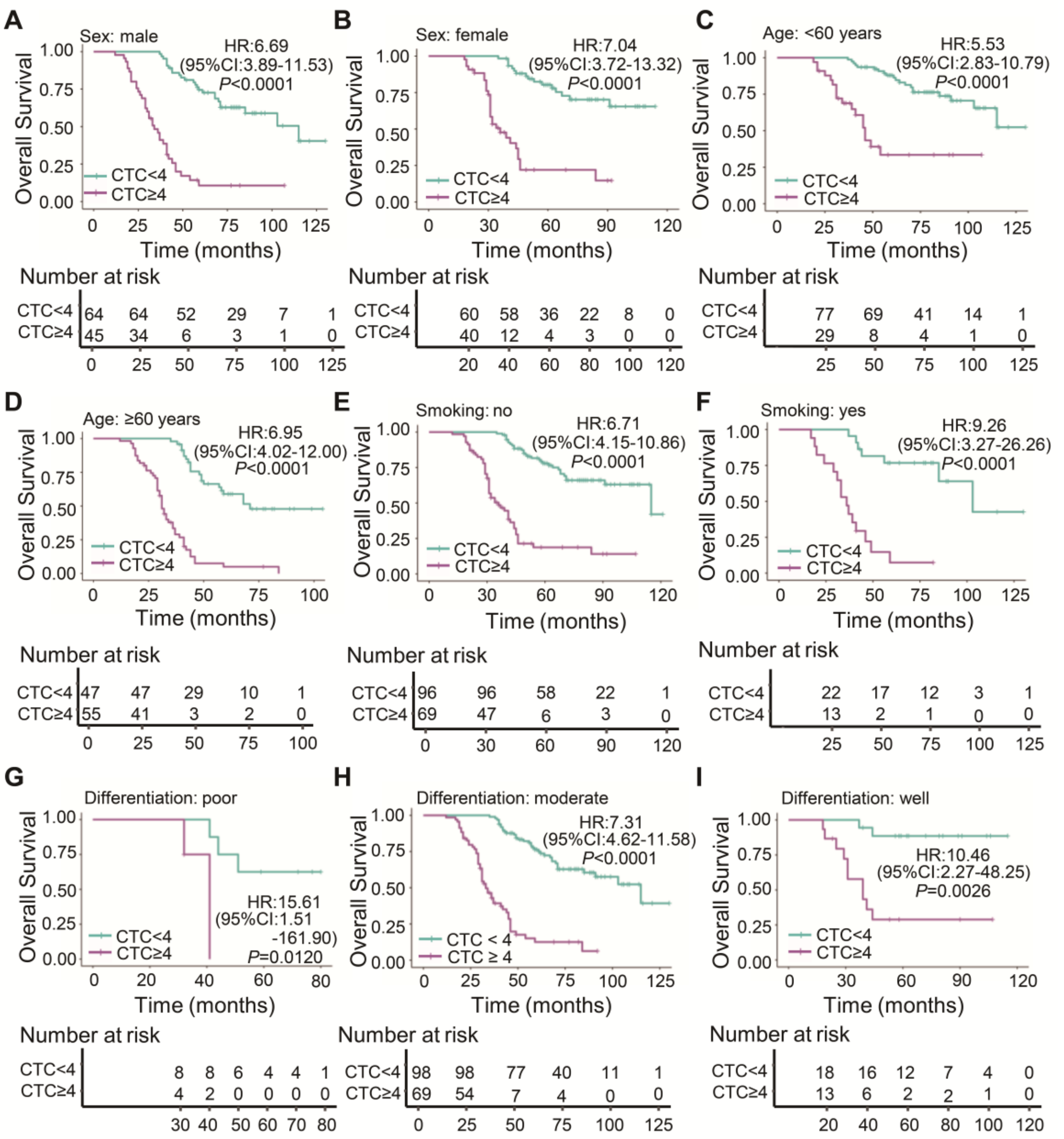

Supplementary Figure S8. Kaplan-Meier curves for predicting OS with different clinicopathological characteristics according to the cutoff value of 4 CTCs per 7.5 mL of blood in all 212 patients. A, male with CTC<4 or CTC≥4 groups; B, female with CTC<4 or CTC≥4 groups; C, age<60 with CTC<4 or CTC≥4 groups; D, age≥60 with CTC<4 or CTC≥4 groups; E, smoking with CTC<4 or CTC≥4 groups; F, no smoking with CTC<4 or CTC≥4

groups; G, poor differentiation with CTC<4 or CTC≥4 groups; H, moderate differentiation with CTC<4 or CTC≥4 groups; I, well differentiation with CTC<4 or CTC≥4 groups. CTC indicates circulating tumor cell; OS, overall survival; HR, hazard ratio.

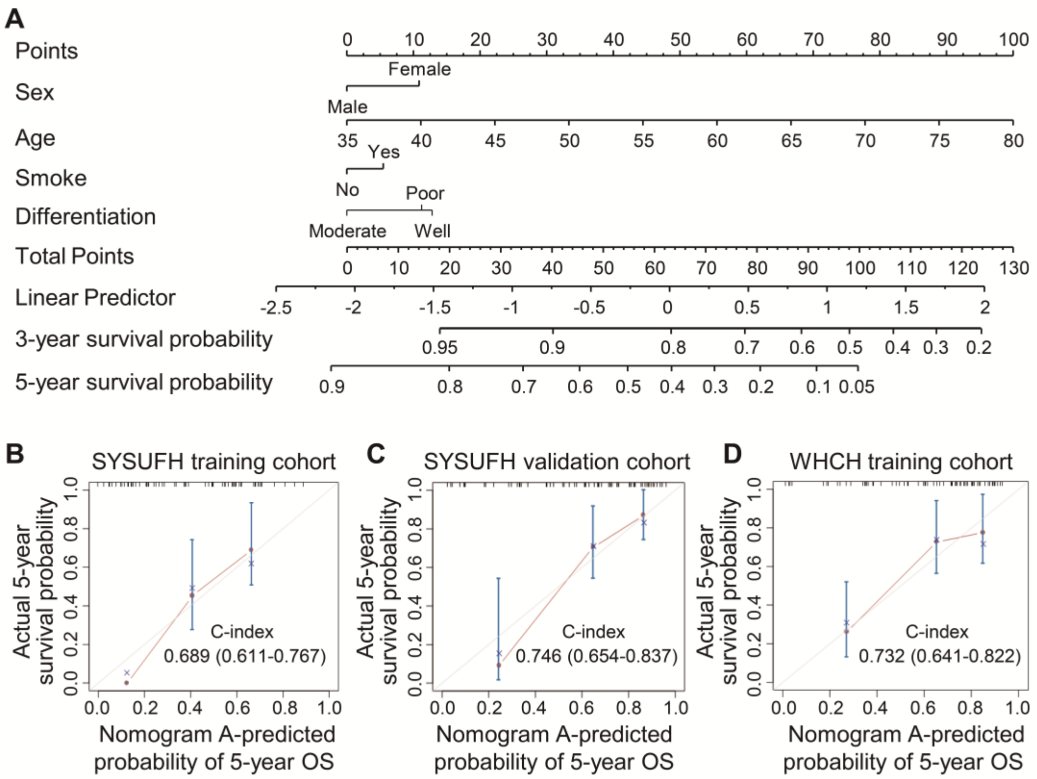

Supplementary Figure S9: Nomogram A (excluding CTC level) for predicting the 3- and 5-year survival probabilities of patients with stage IB LUAD and calibration curves for testing the stability of nomogram. A, Nomogram A was based on the multivariate analysis results of the SYSUFH training cohort. B, Calibration curves of the SYSUFH training cohort. C, Calibration curves of the SYSUFH validation cohort. D, Calibration curves of the WHCH validation cohort. CTC indicates circulating tumor cell; C-index, concordance index; SYSUFH, First Affiliated Hospital of Sun Yat-sen University; WHCH, Wuhan Central Hospital; LUAD, lung adenocarcinoma.
